# LncRNAs and immunity: watchdogs for host pathogen interactions

**DOI:** 10.1186/s12575-017-0052-7

**Published:** 2017-04-27

**Authors:** Peerzada Tajamul Mumtaz, Shakil Ahmad Bhat, Syed Mudasir Ahmad, Mashooq Ahmad Dar, Raashid Ahmed, Uneeb Urwat, Aadil Ayaz, Divya Shrivastava, Riaz Ahmad Shah, Nazir Ahmad Ganai

**Affiliations:** 1 Division of Biotechnology, Faculty of Veterinary Sciences and Animal Husbandry, SKUAST-K, Shuhama, Srinagar, Jammu and Kashmir India; 2 Division of Animal Breeding and Genetics, Faculty of Veterinary Sciences and Animal Husbandry, SKUAST-K, Shuhama, Srinagar, Jammu and Kashmir India; 3 School of Life Sciences Jaipur National University, Jaipur, Rajasthan India

**Keywords:** LncRNA, Immunity, Practical miscellany, Immunogene expression host-pathogen interaction

## Abstract

Immune responses combat various infectious agents by inducing inflammatory responses, antimicrobial pathways and adaptive immunity. The polygenic responses to these external stimuli are temporally and coordinately regulated. Specific lncRNAs are induced to modulate innate and adaptive immune responses which can function through various target interactions like RNA-DNA, RNA-RNA, and RNA-protein interaction and hence affect the immunogenic regulation at various stages of gene expression. LncRNA are found to be present in various immune cells like monocytes, macrophages, dendritic cells, neutrophils, T cells and B cells. They have been shown to be involved in many biological processes, including the regulation of the expression of genes, the dosage compensation and genomics imprinting, but the knowledge how lncRNAs are regulated and how they alter cell differentiation/function is still obscure. Further dysregulation of lncRNA has been seen in many diseases, but as yet very less research has been carried out to understand the role of lncRNAs in regulation during host-pathogens interactions. In this review, we summarize the functional developments and mechanism of action of lncRNAs, in immunity and defense of host against pathogens.

## Background

The assumption that ncRNAs can play a critical role in various biological processes has been recognized for a long time such as rRNAs and tRNAs are required for protein synthesis [1, 2] and small nuclear RNAs (snRNAs) and small nucleolar RNAs (snoRNAs) play a major role for mRNA splicing and nuclear organization [3]. Recent developments in sequencing technologies have revealed that ncRNA transcription is more prevalent than previously appreciated [4, 5]. A major breakthrough in molecular biology over the last two decades has been the discovery and demonstration of function for lncRNAs. The emerging role of lncRNAs is only now starting to be cataloged. The major roles of lncRNAs are being uncovered in a diverse array of processes of genomic imprinting to X chromosome inactivation (Xist), to stem cell differentiation, to cancer metastasis and immunity and much more. In our previous review [6], we comprehensively describe the molecular functions and mechanisms of various lncRNAs. The sequencing technologies revealed their natural structure and precisely determined what type of interaction they follow, for example, RNA-RNA, RNA-DNA, or RNA-Protein interactions. Long noncoding RNAs produce diverse processes to regulate gene expression through transcription, splicing, nucleic acid degradation, decoy, and translation. The emerging role of lncRNAs in immune responses became a subject of attraction with a breakthrough study, which reported that lncRNAs might regulate the innate immune response [7]. Since then, many lncRNAs have been functionally characterized through microarrary and RNA-Seq techniques with respect to innate immunity. This provided a new insight into the role of lncRNAs in immune system regulation. Since then a large number of lncRNAs were discovered, such as Lethe, PACER, THRIL, and NEAT1, representing a new class of molecules that is implicated in regulating the immune gene expression [8] and immune cell functions [9, 10].

Importance of lncRNAs is emerging for their regulatory role in physiological and pathological responses [11, 12]. Their functional utility in the immune response is quickly emerging and this is actually the subject of this review. In forthcoming sections, we provide the basis for the subsequent functional and mechanistic analysis of individual lncRNAs under the headings of immunity and host pathogen interaction.

### Practical miscellany of Immune-related lncRNAs

The role of lncRNAs in immune regulation is in its infancy and is becoming the areas of concern in diverse research areas. Recent studies reveal that various lncRNAs are present in immune cells including monocytes, macrophages, dendritic cells, neutrophils, T cells and B cells. The expression levels of lincRNA have been shown to be associated with development, differentiation and activation of immune cells [13]. With a wealth of information coming from different publications regarding immune-related lncRNAs, it is worth mentioning the functional diversity of these lncRNAs. Currently, many of the reported immune-related lncRNAs are located close to or overlapping with immune-related protein coding gene clusters, such as IL1 -RBT46 [14], lnc-IL7R [15, 16] and lincRNA-Ccr2-5′ AS [17] [Table 1]. These are found to regulate their adjacent protein coding genes in cis or in trans-acting manners. Moreover, recent reports show that the regulatory functions of many immune-related lncRNAs are mostly involved in processes of RNA/protein binding or RNA/DNA base-pairing [18]. Given the vast number of interactions discovered, immune-related lncRNA can interact with transcription factors and signaling molecules (NF-κB, STAT3) [19–21], RNA binding proteins (hnRNP, HuR), [18, 22, 23] as well as chromatin remodeling components (PRC2, WDR5) [24, 25]. Nonetheless, further understanding of immune-related lncRNA functions and their underlying molecular mechanisms will undoubtedly shed more light on our knowledge about how lncRNAs function in immune regulation.

**Table 1:** Functions of immune related lncRNAs

S. No.	lncRNA	Function	Reference
1	Hotair	Promotes cancer metastasis and progression via epigenetic variations in the chromatin state	[31]
2	Lnc-IL7R	Epigenetically regulates inflammation	[15]
3	NRON	Transcription regulator for immune regulation	[33]
4	NeST/Tmevpg1	Epigenetically regulates the adaptive immunity through IFN-gamma	[24, 34, 35]
5	IL1β	Chromatin modulation.	[14]
6	IL1β-RBT46	Regulates the homeostasis of IL-1β in monocytes	[14]
7	IL1b-eRNA	Expression of proinflammatory mediators e.g., CXCL8 and IL-1β	[14]
8	PACER	Involved in multiple processs related to regulation of immunogene expression	[21]
9	LincRNA-COX2	Role in TLR-induced expression of interleukin-6	[7, 22]
10	Lnc-DC	Required for the differentiation of monocytes to dendritic cells	[20]
11	Lethe	Upregulated during inflammation	[19]
12	THRIL	Regulate expression of tumour necrosis factor (TNF) in human monocytes	[10, 23]
13	PAN	epigenetically regulates viral gene expression and promotes the switch from latent to lytic infection	[52, 54]
14	NRAV	Modulation of transcription of multiple interferon-stimulated genes (ISGs) i.e,. MxA and IFITM3	[55]
15	NEAT1	Triggers transcriptional activation of IL-8 in response to viral infection	[10]

### LncRNAs and modulation of immunogenic expression

Besides, lncRNA regulate transcription via chromatin modulations [26], several lncRNAs have been found to target directly or indirectly on specific transcriptional factors [8]. More recently, specific type of lncRNAs like enhancer RNA (eRNA) have been reported to modulate target gene expression [6, 27]. Here we discuss several immune regulatory lncRNAs that modulate gene transcription through their unique mechanisms.

#### HOTAIRM1

HOX antisense intergenic RNA myeloid 1 (HOTAIRM1) is enciphered in the human HOXA gene cluster and is associated with the maturation of granulocytes [28] and is a key regulator of HOXA genes which are involved in the transcriptional regulation of acute myeloid leukemia (AML) [29, 30] and normal hematopoiesis [31]. HOTAIRM1 is specifically expressed in myeloid cells, and is upregulated in retinoic acid induced normal human hematopoietic stem cells. Knockdown of HOTAIRM1 in the NB4 acute promyelocytic leukemia cell line blunts retinoic acid induced expression of HOXA1 and HOXA2 (but not distal HOXA genes) as well as CD11b and CD18 genes which are involved in myeloid differentiation, resulting in retarded all-trans retinoid acid (ATRA)-induced granulocytic differentiation and significantly larger population of immature and proliferating cells.

#### Lnc-IL7R

A novel lncRNA viz, lnc-IL7R identified from LPS-stimulated human monocytic THP-1 cells are transcribed from the 30 UTR of IL-7R gene in the sense orientation and the expression of lnc-IL7R was found to be upregulated in LPS stimulated monocytic THP-1 cells [15] and human peripheral blood mononuclear cells (PBMNC). Lnc-IL7R has also been studied to negatively regulate expression of IL-6, IL-7R, IL-8, VCAM-1 and E-selectin [15]. Furthermore, a study revealed that lnc-IL7R knockdown decreased the trimethylation of histone H3K27 at promoters of inflammatory mediators, suggesting that lnc-IL7R epigenetically regulates inflammatory responses [15].

#### NRON

NRON is non-coding repressor of NFAT (Nuclear Factor of Activated T cells), first identified during a short hairpin RNA (shRNA) library screening against 512 evolutionarily conserved lncRNAs [32]. NFAT is a highly phosphorylated transcriptional factor present in the cytoplasm of resting cells. NFAT is dephosphorylated and transported from the cytoplasm into the nucleus in response to calcium-dependent signals, to induce expression of target genes such as IL-2 (see Fig. 2). It has been found that heavily phosphorylated NFAT is located within a cytoplasmic RNA-protein complex that contains lncRNA NRON, IQGAP1 (a scaffold protein) and three NFAT inhibitory kinases, dual-specificity tyrosine phosphorylation-regulated kinase (DYRK), Casein kinase 1 (CK1), and Glycogen synthase kinase 3 (GSK3) [33]. Furthermore, it was confirmed that knockdown of lncRNA NRON results in nuclear accumulation of NFAT [32], suggesting that NRON acts as a transcription repressor by inhibiting nucleocytoplasmic shuttling of NFAT. Conclusively, it came into sight that lncRNAs such as NRON can function as a transcriptional regulator for immune regulation.

#### NeST/Tmevpg1

Nest (Nettoie Salmonella pas Theilers’s), also called Tmevpg1, is a long noncoding RNA gene located downstream adjacent to the IFN γ-encoding gene and transcribed convergently to the IFN-γ gene in both humans and mice [34]. In the mice, NeST RNA contains six exons spread over a 45 kb stretch [34]. The most common splice variant (914 nucleotides long), is expressed in natural killer cells, CD4+ T cells and CD8+ T cells and has no AUG codons in translational discourse that appear functional. The location and orientation of human NeST are conserved, but the primary transcript includes the opposite strand of the entire IFNγ gene. Its expression has been found to be linked with IFN-γ expression and is induced in response to the Th1-differentiation program by mechanisms relying upon T-bet and STAT4 [24, 34, 35]. Mice over expressing NeST shows increased susceptibility to Theiler’s virus persistence, but remarkable resistance to Salmonella pathogenesis. Mechanistic analysis indicated its interaction with WDR5, a core subunit of the histone H3K4 methyltransferase complex, leading to modification of H3 methylation at the IFN-γ locus, thereby epigenetically regulating IFN-γ expression [24, 35]. A recent report revealed that T-bet guides epigenetic remodeling of lncRNA NeST distal and proximal enhancers in developing and differentiated effector Th1 cells, leading to the recruitment of stimulus-inducible transcription factors (e.g., NF-κB and Ets-1), to the locus to achieve Th1-lineage-specific expression of IFN-γ [36]. Thus, it appears that NeST regulates T cell function through multiple mechanisms.

#### IL1b-eRNA, IL1b-RBT46, antisense transcript of IL1β and IL1-α

Recent studies revealed multiple non-coding transcripts that are located close to the IL-1β gene, including antisense-transcript of IL-1β (anti-IL1β transcript), IL1β-RBT46 and IL1β-eRNA, [14, 37]. The anti-IL1β transcript and IL-1β gene are in nip and tuck positions. Moreover, the non-coding anti-IL-1β is transcribed from the 5′ upstream promoter sequence of the coding gene IL-1β [37]. In mouse macrophages, the anti-IL1β expression is effectively regulated during LPS-induced macrophage activation [37]. The ectopic over expression of anti-IL1β transcript significantly suppressed LPS-induced IL-1β expression in RAW264.7 cells [37]. The anti-IL1β transcript modulates the chromatin structure surrounding IL-1β promoter by decreasing H3K4 trimethylation [37]. Together, antisense IL-1β seems to act as a natural antisense transcript of IL-1β gene to regulate the homeostasis of IL-1β in cells.

Recently, a large number of long non-coding RNAs including 65 antisense lncRNAs, 76 enhancer RNAs (eRNAs), 40 canonical lncRNAs, and 35 regions of bidirectional transcription (RBT) in human monocytes were identified after the stimulation with LPS [14]. Interestingly, genomic region flanking inflammatory cytokine IL-1β gene displays high transcriptional complexity. Situated within IL-1β locus, a downstream eRNA, namely, IL1β-eRNA and an upstream mRNA-surrounding RBT called IL1β-RBT46 have been identified [14]. Further studies revealed the predominant localization of both IL1β-eRNA and IL1β-RBT46 in the nucleus of naive and LPS-stimulated cells. The expression of IL1β-eRNA and IL1β-RBT46 is mediated by NF-κB, (a classical proinflammatory transcription factor) [14]. Knockdown of IL1β-eRNA and IL1β-RBT46 in human monocytes selectively attenuates the LPS-induced expression of proinflammatory mediators including CXCL8 and IL-1β through unknown mechanisms [14]. Given the genomic position of these lncRNAs and eRNAs, they are considered to function as essential regulators of the immune response [38], while the underlying mechanisms remain to be elucidated. Beyond IL-1β, the IL-1α expression is also regulated by lncRNAs: Antisense IL-1α (AS IL-1α) RNA shows similar expression patterns with the IL-1α protein-coding gene, with which it partially overlaps [39]. Using loss-of-function shRNA approaches, AS IL-1α was shown to be essential for IL-1α gene transcription. Knockdown of AS IL-1α by RNA interference compromised the recruitment of RNAPII to the promoter of IL-1α and as a result decreased levels of IL-1α mRNA in macrophages exposed to LPS [39]. Like many inflammatory genes, a substantial number of lncRNAs are only expressed in innate immune cells following their activation, while other lncRNAs that are abundantly expressed are down regulated when cells are exposed to inflammatory stimuli [22].

### LNC RNAs and modulation via interacting with proteins

LncRNAs physically interact with transcription factors, structural proteins, and RNA binding proteins (RBPs), which in turn contribute to regulate the activity and function of these molecules [18]. Besides the regulation of a gene transcription, lncRNAs can also act at the protein level [22]. They can function as scaffolds for protein complex and coordinate the gene expression at the post - transcriptional level [6, 19]. Here, we provide the detail of some lncRNAs regarding to this notion in the immune system.

#### PACER

**Fig. 1:**
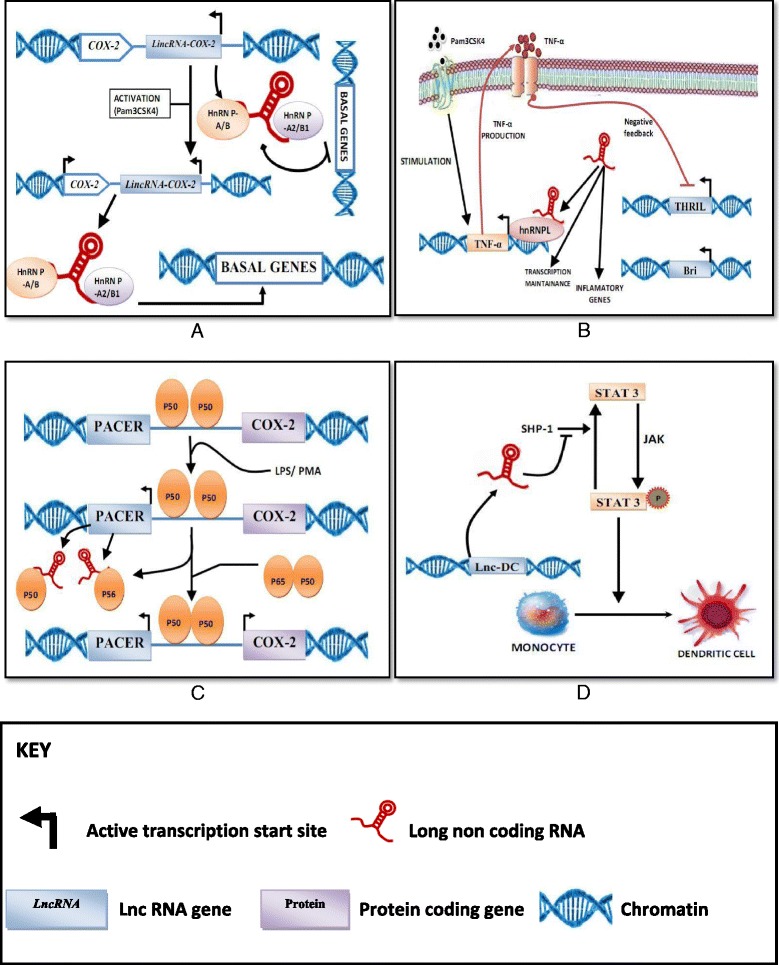
LncRNAs in the immune response (**a**) LincRNA-COX-2 is localised 3′ of the COX2 gene and shows expression on stimulation of Pam3Csk4 in bone marrow-derived macrophages of mouse. It has widespread effects on expression of inflammatory genes, transcription repression of anti-inflammatory genes in non-stimulated cells and enhances the expression of proinflammatory genes on exposure of Pam3Csk4 through an interaction with hnRNP-A/B and hnRNP-A1/B1 [7, 22]. **b** THRIL was identified as an antisense lncRNA using human THP1 macrophages (overlapping BRI3BP) that enhances transcription of TNF by constituting a complex with hnRNPL and binding to the promoter of TNF [10, 23]. THRIL shows basal expression; but, the expression is decreased in a negative feedback loop after the induction of TNF-α release by Pam3Csk4 [10, 23]. THRIL also has a regulatory role in basal and Pam3Csk4-induced gene expression. **c** PACER is localised upstream of the Cox2 transcriptional start site and is shows expression in the antisense direction. PACER induced COX2 expression by inhibiting the repressive action of the p50 homodimer (of NF-kB) bound at Cox2 promoter site [21] (**d**) Lnc-DC expression is essential for differentiation of human monocytes into dendritic cells. Lnc-DC promotes STAT3 phosphorylation via inhibiting the action of Src homology region 2 domain- containing phosphatase-1 (SHP-1) [20]

PACER (p50-associated Cox2 extragenic RNA) is a well-known lncRNA located upstream of the Cox2 transcriptional start site and expressed in the antisense direction in humans. The PACER homolog in mice has been identified as cyclooxygenase II enzyme-divergent (Ptgs2os) whose expression in mouse embryonic fibroblasts is highly induced by LPS, proinflammatory cytokines (IL-1β and TNF) and various TLR agonists viz., Pam3CK4, HKLM, Poly(I:C). Interestingly, Cox2-divergent displays similar upregulated expression patterns upon the cytokine/TLR agonist stimulations in RelA/MEFs as compared to wild type MEFs, suggesting indirect regulation of lncRNA Cox2-divergent by RelA (NF-κB component) [19]. Moreover, Krawczyk and Emerson reported the expression of lncRNA Cox2-divergent homolog PACER in primary human mammary epithelial cells (HMECs) and in human monocytes that are in the process of macrophage differentiation. They also revealed the regulatory role of PACER in COX-2 gene expression [21]. Furthermore, PACER is also been suggested to be involved in regulation of NF-κB signaling by physically interacting with NF-κB p50 thereby sequestering the transcription factor binding to the promoters of target genes such as COX2 (see Fig. 1c) [21]. The sequestration of transcriptional factor facilitates the recruitment of histone acetyltransferase p300 and assembly of RNA polymerase II pre-initiation complex at the promoter of COX2 gene. PACER expression is induced by chromatin boundary/insulator factor CCCTC-binding factor (CTCF), which in turn forms a permissive chromatin environment in the upstream region of COX2 gene [21]. All together, these studies show the involvement of PACER lncRNA in multiple processes related to regulation of immunogene expression.

#### LincRNA-Cox2

LincRNA-Cox2 is located 51 kb upstream of human cyclooxygenase 2 gene (COX2, also known as prostaglandin-endoperoxide synthase 2 or Ptgs2) and is an important component of inflammatory response. The impact of lincRNA-Cox2 on the TLR response is broad-acting with unprecedented effects (see Fig. 1a). Silencing of lincRNA-Cox2 does not alter expression of Cox2 (Ptgs2), but causes an increase in expression of several immune responsible genes in resting macrophages, including IFN-stimulated genes (ISGs) (Oas1a, Irf7, Ifi204, Oas1l, Oas2, and Isg15, chemokines (Cl3cl1,Ccl5,) and chemokine receptors (Ccrl). The LincRNA-Cox2 expression is remarkably induced in dendritic cells and macrophages challenged with microbial pathogens and various TLR ligands such as Pam3CSK4, LPS and R848 in MyD88 and NF-κB dependent manner [7, 22]. Recently a study revealed that lincRNA-Cox2 is essential for the induction of other immune-related genes, such as Tlr1, IL-6, and IL-23a in macrophages derived from bone marrow by Pam3CSK4 treatment (see Fig. 1a) [22]. Thus, it appears that lincRNA-Cox2 plays a role in, either activation or repression of immune-regulatory gene expression in macrophages. Previously, lincRNA-Cox2 is shown to have transcriptional repressive functions via interacting with heterogeneous nuclear ribonucleoprotein (hnRNP) A/B and A2/B1 (see Fig. 1a) [22]. On the other hand, lincRNA-Cox2 was shown to facilitate the inducible expression of a distinct cluster of immune response genes, including proinflammatory cytokines and other inflammatory mediators. In addition to its role in macrophages, lincRNA-Cox2 is also regulated downstream of NF-κB in epithelial cells [40]. Similar to what was observed in macrophages, knockdown of lincRNA-Cox2 resulted in reprogramming of the gene expression profile in intestinal epithelial cells exposed to TNF-α. In particular, lincRNA-Cox2 appears to repress the transcription of IL-12b, and mediates these effects via its interactions with the Mi-2 nucleosome remodeling and deacetylase (Mi- 2/NuRD) repressor complex, which this lincRNA appears to guide to the Il12b promoter region [40]. These data provide mechanistic insight into the role of lincRNA-Cox2 in promoting epigenetic modulation of cytokine genes and identify lincRNA-Cox2 as a novel regulator of both macrophage and intestinal epithelial inflammatory responses. The Ptgs2 (Cox2) genomic locus in mice encodes a second lncRNA called Cox2-divergent (Ptgs2 opposite strand; Ptgs2os) [19, 22]. This Cox2-divergent lncRNA is located at the 5-end of Ptgs2 (nonoverlapping), and is transcribed on the opposite (negative) DNA strand [19]. Although the functions of Cox2-divergent remain to be further clarified, it is highly inducible in mouse embryonic fibroblasts exposed to TNF-α and LPS [19]. The RNA binding protein family of hnRNPs includes many multifunctional proteins with a regulatory role in the gene expression and the post transcriptional modification [41]. With the availability of lincRNA-Cox2 knockout mouse [42], further research is required to clarify the mechanisms of lincRNA-Cox2 in the immune responses.

#### Lnc-DC

Dendritic cells (DCs) are antigen-presenting cells, which links innate immune system with adaptive immune system. Recently, a genome wide screening study revealed a cohort of lncRNAs which are differentially expressed during development of human DCs. Among these lnc-DC has been revealed to act as an inducer during DC differentiation [20]. PU.1 a promoter binding transcription factor is shown to control lnc-DC transcription, suggesting its regulatory role in lnc-DC gene expression. Meanwhile, H3K4me3 and H3K27ac are found to activate histone modifications on lnc-DC loci, thereby forming an accessible chromatin structure and hense facilitating an exclusive expression of lnc-DC in human DCs [20]. Knockdown of lnc-DC impaired differentiation of DCs from human monocytes in vitro and from mouse bone marrow cells in vivo and reduced capability of DCs to stimulate T cell activation (see Fig. 1d). Lnc-DC, was shown to be upregulated during human DC development and was found to be highly expressed in Lin − MHCII + CD11c + conventional DCs, but absent from plasmacytoid DCs, monocytes, or other leukocyte subsets [20]. Lentiviral knockdown of lnc-DC during human DC development was shown to have broad functional consequences, including impaired expression of surface receptors critical for T-cell activation (CD80/86, HLA-DR, CD40), impairment in antigen uptake by monocyte-derived DCs, and decreased IL-12 production after stimulation with LPS. lnc-DC mediated these effects by activating STAT3 (signal transducer and activator of transcription 3, a transcription factor. Mechanistically, it was shown to regulate STAT3 activity, a critical regulator of DC maturation. lnc-DC bound directly to STAT3 in the cytoplasm, preventing STAT3 binding to and dephosphorylation by SHP1. This resulted in maintenance of STAT3 phosphorylation in the presence of lnc-DC. It is worth mentioning that a later study suggested that murine lnc-DC actually encodes a small secreted protein called Wdnm1-like [43], and therefore further studies are required to better understand the role of lnc-DC in murine DC differentiation.

#### Lethe

lncRNA Lethe is a Rps15a pseudogene (Rps15a-ps4) and was first identified as a functional pseudogene via genome wide sequencing of TNF-α stimulated mouse embryonic fibroblasts. Lethe has recently been revealed to be localized in chromatin and is suggested to function as a negative regulator of NF-κB by binding to RelA (p65), resulting in the inhibition of RelA, thence regulating the NF-κB target gene expressions, such as IL-8, IL-6 and SOD2 [19]. Lethe is markedly upregulated in response to stimulation with glucocorticoid receptor agonists such as dexamethasone, proinflammatory cytokines such as IL-1β, and TNF-α but the expression of Lethe is not responsive to TLR agonist challenges [19]. Therefore, Lethe functions as a decoy lncRNA and is a negative feedback inhibitor of NF-κB signaling in inflammation.

#### THRIL

THRIL (TNF and heterogeneous nuclear ribonucleoprotein L related immunoregulatory lincRNA) has been recently discovered via a custom microarray of the activated THP1 monocytes. It has been studied that THRIL expression is involved with inflammation in Kawasaki disease [23]. Recently a number of differently expressed lncRNAs associated with activation of cells by Pam3CSK, a TLR2 ligand were discovered by using differentiated human macrophage-like THP1 cell model [23]. Among them, THRIL is significantly downregulated in response to the stimulation. Moreover, THRIL is shown to mediate the effect of Pam3CSK4 on induction of expression of CSF1, TNFα, IL-8, IL-6, CXCL10 and CCL1 suggesting its role in immune regulation [23]. Additionally, THRIL is found to interact with heterogeneous nuclear ribonucleoprotein L (hnRNPL). The THRIL-hnRNPL complex binds to TNFα promoter thereby regulating its transcription in both basal and Pam3CSK4-activated conditions. Interestingly, the THRIL expression can be inhibited by TNFα (see Fig. 1B) [23]. THRIL loss-of-function (shRNA) studies revealed that THRIL contributes to the inducible expression of the proinflammatory cytokine mediators TNF-α and IL-6 upon Pam3CSK4 stimulation [44]. Further supporting role for THRIL in immune gene regulation, chromatin immunoprecipitation (ChIP) experiments indicated that heterogenous ribonucleoprotein (hnRNP)-L localized to the TNF-α promoter upon Pam3CSK4 stimulation. A very different mechanism by which lncRNAs can induce inflammatory responses seems to be a direct inflammatory response directed against the ssRNA itself. This has recently been demonstrated by transfection of in vitro transcribed lncRNAs into myeloid cells, which led to a strong induction of proinflammatory cytokines such as IL-6, IL-12, or TNF-α [45]. Therefore, THRIL is a novel negative feedback regulator for termination of TNFα expression in inflammatory response. The role of THRIL in TNFα expression marks the significant regulatory role of lncRNA immune-related gene expression [10].

#### Rmrp

Rmrp is a highly conserved single exon-transcript of 268 nucleotides, which has been shown to be mutated in patients with cartilage hair dysplasia, a disease with a broad clinical spectrum including skeletal dysplasia and immunodeficiency. T helper 17 (TH17) lymphocytes protect mucosal barriers from infections, but also contribute to multiple chronic inflammatory diseases. The identified Rmrp-DDX5-RORγt complex, which has been shown to interact with key genomic sites to promote TH17 function, could represent a new therapeutic target for autoimmune diseases. The differentiation of Th17 cells is controlled by the nuclear hormone receptor (Retinoic acid receptor-related Orphan nuclear Receptor gamma t, RORγt), which drives TH17 cell differentiation and effector functions by controlling expression of interleukin IL-17a, IL-17f, and IL-22, amongst others. A study in understanding the mechanism by which RORγt controls transcription of these target genes in Th17 cells, RORγt-interacting proteins were identified and called as DEAD-box RNA helicase DDX5. DDX5 is required for induction of a subset of RORγt target genes. Importantly, DDX5 mediated these effects by interacting with an lncRNA called Rmrp [46]. Mice carrying a single point mutation in Rmrp, corresponding to the mutation in cartilage hair dysplasia patients, had severely impaired binding to DDX5 and RORγt-compromised transcription of multiple RORγt target genes. The discovery of Rmrp as a functional partner of DDX5 and a regulator of RORγt-dependent TH17 cell effector functions reveals a new layer of complexity in TH17 cell function [47].

### Long non-coding RNAS in host-pathogen interaction

**Fig. 2:**
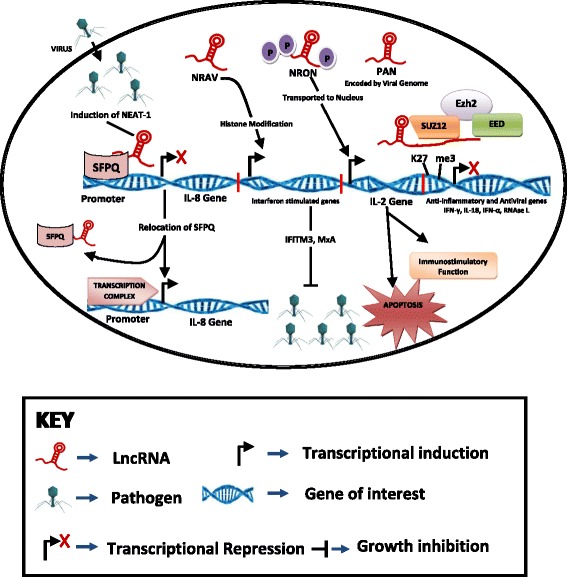
Different types of pathogens, including viruses infect the host and then induce functional lncRNAs in host, which have been studied to control and modulate the pathogen infections. The viral lncRNA PAN suppresses expression of host genes involved in the inflammatory and antiviral responses, including IFNγ, IL-18, IFNA16, and RNase L [61]. A recent report showed that PAN can physically interact with polycomb group proteins, such as PRC2 and mediate repression of host cellular gene expression [25]. On the other hand, lncRNA NRAV also has an inhibitory role in initial transcription of multiple interferon-stimulated genes (ISGs), such as MxA and IFITM3, via epigenetically regulating histone modifications of these genes [53]. NEAT1 is shown to bind to SFPQ (a paraspeckle protein) and play an important role in formation of nuclear paraspeckle body. Recently, a study demonstrated that SFPQ silences IL-8 expression via binding to IL-8 promoter in normal physiological states [51]. NFAT is a highly phosphorylated transcriptional factor present in the cytoplasm of resting cells. NFAT is dephosphorylated and transported from the cytoplasm into the nucleus in response to calcium-dependent signals, to induce expression of target genes such as IL-2, which plays a key role in enduring cell-mediated immunity [33]

So far the understanding of the mechanistic role of lncRNAs in or following infection and host responses to infection, poor and limited to a few studies and even more so, primarily to four models only. Each of these reports show an interesting and intriguing new opportunity to understand and evaluate the function and interaction of long non-coding RNA following infection. Mechanistically, dysregulation of long ncRNAs could control downstream regulation of genes at several functional levels stretching from epigenetic changes influencing chromatin organization to post-transcriptional regulation at transcript levels as well as via direct interaction with other biomolecules such as proteins and RNAs [46, 48]. These interactions could affect (a) host responses to a pathogen not excluding immunological mechanisms (b) regulation of growth and replication of pathogen (c) regulation of apoptosis or survival (d) general stress responses. While there in not certainty about the exact mechanism through which viral lncRNAs act, it has been suggested that the viral long ncRNAs exploit the interaction networks within hosts, thereby influencing their response to infections in an attempt to evade the immunological response. A variety of mechanisms are employed in doing this, besides the inhibition of the RNAi response [49]. The mechanism through which long ncRNAs mediate its mechanism in host-pathogen interactions have been summarized and schematically drawn in Fig. 2. The immune system plays an important role in defense against microbial pathogens. Recently, a class of host-encoded lncRNAs such as NEAT1 and NRAV has been identified to play a functional role in controlling the host immune responses upon microbial invasion [50]. On the other hand, some microbial species can produce lncRNAs that play pivotal roles in pathogen life cycles as well as affecting host-pathogen interactions. In consideration, the lncRNA-mediated regulation of host-pathogen interactions during pathogen invasion has also emerged. Here, we highlighted the following lncRNAs in this category.

### PAN

Recent studies revealed that pathogens can also express functional lncRNAs. One of well-characterized pathogen/microbial-derived lncRNAs is PAN RNA (polyadenylated nuclear RNA) [51, 52]. Kaposi’s sarcoma-associated herpesvirus (KSHV) genome encodes the PAN lncRNA where it is implicated in the KSHV viral gene expression and replication [53]. PAN interacts with demethylases UTX and JMJD3 thereby recruiting histone-modifying complexes to the KSHV genome. Thus PAN epigenetically regulates viral gene expression and promotes the switch from latent to lytic infection [52, 54]. On the other hand, PAN RNA has a regulatory role in host immunity. The viral lncRNA PAN suppresses expression of host genes involved in the inflammatory and antiviral responses, including IFNγ, IL-18, IFNA16, and RNase L [52] (see Fig. 2). A recent report showed that PAN can physically interact with polycomb group proteins, such as PRC2 and mediate repression of host cell gene expression [25]. Taken together, PAN is a multifunctional viral lncRNA involved in regulation of both viral and host gene expression.

#### NRAV

NRAV (negative regulator of antiviral) is recently discovered as a and has a key role in regulation of antiviral innate immunity via a genome-wide profiling of lncRNA in influenza virus A/WSN/33 (H1N1) infected human alveolar epithelial A549 cells [55]. The down-regulation of lncRNA NRAV is considered to be associated with infections by numerous viruses, including ssRNA virus such as influenza A,Sendai virus (SeV) and virus (IAV), dsRNA virus such as Muscovy Duck Reovirus (MDRV), and DNA virus such as herpes simplex virus (HSV). Moreover, NRAV is found to modulate virus replication, production and virulence. On the other hand, lncRNA NRAV also has an inhibitory role in the initial transcription of multiple interferon-stimulated genes (ISGs), such as MxA and IFITM3, via epigenetically regulating histone modifications of these genes [55] (see Fig. 2). Together, normally lncRNA NRAV seems to play a role in controlling ISG expression. Upon the viral infection, the reduction of NRAV could boost the host innate immune response through accumulating anti-viral proteins (such as ISGs), thus facilitates the virus clearance.

#### NEAT1

NEAT1 (nuclear enriched abundant transcript 1 or nuclear paraspeckle assembly transcript 1) was first identified as an inducible nuclear lncRNA in mouse brain infected with Japanese encephalitis virus or Rabies virus [56]. Later, it was found that NEAT1 can be dramatically induced in HIV-1 infected T cells as well as influenza virus and herpes simplex virus infected epithelial cells [10, 57]. Moreover, treatment with TLR3 ligand poly I:C mimics the effect of viral infection on stimulation of lncRNA NEAT1 expression [58]. In addition, NEAT1 is shown to bind to SFPQ (a paraspeckle protein) and play an important role in formation of nuclear paraspeckle body. Recently, a study demonstrated that SFPQ silences IL-8 expression via binding to IL-8 promoter in normal physiological states [58] (see Fig. 2). In response to viral infection, induction of NEAT1 results in relocation of SFPQ from the IL-8 promoter to paraspeckles followed by triggering transcriptional activation of IL-8 [10]. In addition, NEAT1 can regulate HIV-1 replication through affecting the nucleus-to-cytoplasm export of Rev-dependent instability element (INS) containing HIV-1 mRNA [57]. Taken together, lncRNA NEAT1 plays an important role in the innate immune response to viral infection.

### Long Non-Coding RNA expression in response to infection

It has been well recognized that the housekeeping, non-coding RNAs (ncRNAs) are constitutively expressed, whereas many regulatory RNAs, are produced in response to external stimuli and regulate important cellular functions [59–64]. NTT (non-coding transcript in T cells) was found accidentally during activation of human T lymphocytes with phytohemagglutinin or with phorbol 12-myristate 13-acetate and ionomycin [65]. Recently the role of NEAT1, previously known as the Virus Inducible non-coding RNA (VINC1), in the mouse brain infected with the Japanese Encephalitis virus was elucidated and further this study suggested the potential functional consequences of long ncRNAs in infection biology owing to the dysregulation of these ncRNAs during infection processes mostly in response to viral pathogens [66]. Investigative studies on the same long non-coding RNA have emphasized the role of NEAT1 in paraspeckle formation and have been further hypothesized to be an essential component in the host responses to viral infections. Similarly, another long non-coding RNA, viz., Psoriasis susceptibility-related RNA Gene Induced by Stress (PRINS) has been studied to be up regulated after infecting SCID mice with the Herpes Simplex Virus [67] and treatment with bacterial cell wall extracts [68] besides other stress factors, e.g., ultraviolet radiation, etc. PRINS have also been implicated as a conspicuous factor in psoriasis via Genome-wide association studies [67]. Further recently a study reported the distinct signatures of long non-coding RNA expression in a SARS infection model and various dysregulations reposte infection. Similarly the responses were also obtained after treatment with interferons, indicating the likelihood of a common pathway for infection response and lncRNA regulation mediated via the interferon gamma involved immunological pathways [69]. A large number of studies confirmed the association of lncRNAs and immune regulations such as immune responses and infectious diseases. For example, about 20 lincRNAs were shown to be expressed in CD11Cβ bone marrow-derived dendritic cells after being challenged by lipopolysaccharide (LPS), an agonist of the Toll-like receptor 4 [7]. This is the pioneer study to suggest that lncRNAs may play an essential role in the innate immune regulation. Investigators further assess genome-wide differential lncRNA expression patterns associated with inflammation, infection, and differentiation of monocytes into macrophage and dendritic cells, Using microarray and RNA sequencing (RNA-seq) [19, 20, 22, 23, 69–71]. Besides the innate immune responses, increasing evidence revealed the role of lncRNAs in T cell development, differentiation and activation. Using custom microarrays, a study revealed the expression profiles of lncRNAs in mammalian CD^8+^ T cells and revealed hundreds of lncRNAs, expressing in a lymphoid-specific manner and/or changed dynamically during lymphocyte differentiation or activation [72]. Recently, 1524 lincRNA clusters in 42 T cell samples were identified from early T cell progenitors to terminally differentiated helper T cell subsets [17]. The analysis revealed very dynamic and cell-specific expression patterns for lincRNAs during T cell differentiation [17]. In addition to this an another study identified more than 500 novel lincRNAs and described lincRNA signatures in human lymphocytes [68, 73]. Collectively, genome-wide datasets have revealed that lncRNAs emerge as a group of essential molecules that may dynamically regulate the immune system and control immunity.

## Conclusion and future trends

Even with the fast increase in understanding the functional perspectives of lncRNAs, this field is still in its beginning and many questions and challenges are yet to be addressed. Efforts to recognize the role of long noncoding RNAs as key regulators in varied biological processes, especially in host immune responses remains a challenge. Certainly, the advanced technologies and methodologies will continue to clarify how lncRNAs influence diverse biological processes including lncRNA-mediated regulation of host-pathogen interactions, and possibly some unique functions beyond gene expression mechanism. Undoubtedly, in this review we discussed the role of various individual lncRNAs in immune responses. Studying the mechanisms of lncRNAs involvement in host pathogenesis will pave the way for the treatment of many infectious diseases, including cancer.

## Abbreviations

CCTF: CCCTC Binding factor
CK1: Casein kinase 1
DCs: Dendritic Cells
DNA: Deoxyribonucleic acid
DYRK: Dual-specificity tyrosine phosphorylation-regulated kinase
eRNA: Enhancer Ribonucleic acid
GSK3: Glycogen synthase kinase 3.
HOTAIRM1: HOX Antisense Intergenic RNA myeloid 1
HSV: Herpes Simplex Virus
ISGs: Interferon Stimulated Genes
LncRNAs: long non coding Ribonucleic acids
LPS: Lipopolysaccharide
MDRV: Muscovy Duck Reovirus
mRNA: Messenger Ribonucleic acid
NEAT1: Nuclear Enriched Abundant Transcript 1
NFAT: Nuclear Factor Of Activated T-Cells
NRAV: Negative Regulator Of Antiviral
NTT: Non coding Transcript in T-Cells
PAN RNA: Polyadenylated Nuclear Ribonucleic acid
PBMNC: Peripheral Blood Mononuclear Cells
RBP: Ribonucleic acid Binding Protiens
RNA: Ribonucleic acid
rRNA: Ribosomal Ribonucleic acid
SeV: Sendai Virus
shRNA: short hairpin Ribonucleic acid
snoRNA: Small nucleolar Ribonucleic acid
snRNA: Small nuclear Ribonucleic acid
STAT3: Signal Transducer And Activator Of Transcription 3
TMEVPG1: Theiler’s Murine Encephalomyelitis Virus Persistence Candidate Gene 1
tRNA: Transcriptional Ribonucleic acid
VINCI1: Virus Inducible Non Coding Ribonucleic Acid
